# AI-Assisted 3D Intracardiac Echocardiography for Pulsed Field Ablation of Atrial Fibrillation Using a Novel Variable Loop Circular Catheter: A Multicenter Evaluation

**DOI:** 10.3390/jcm14207249

**Published:** 2025-10-14

**Authors:** Antonio Dello Russo, Yari Valeri, Giuseppe Ciconte, Marco Schiavone, Paolo Compagnucci, Antonio Di Monaco, Stefania Riva, Raffaele Salerno, Giovanni Volpato, Laura Cipolletta, Quintino Parisi, Michela Casella, Massimo Grimaldi, Claudio Tondo, Carlo Pappone

**Affiliations:** 1 Department of Biomedical Sciences and Public Health, Marche Polytechnic University, 60126 Ancona, Italy; 2 Cardiology and Arrhythmology Clinic, Marche University Hospital, 60126 Ancona, Italy; 3 Arrhythmia and Electrophysiology Center, IRCCS Policlinico San Donato, 20097 San Donato Milanese, Italy; 4 Department of Arrhythmology, Vita-Salute San Raffaele University, Via Olgettina 58, 20132 Milano, Italy; 5 Department of Clinical Electrophysiology & Cardiac Pacing, Centro Cardiologico Monzino IRCCS, 20138 Milan, Italy; 6 Cardiology Department, Regional General Hospital “F. Miulli”, Acquaviva delle Fonti, 70021 Bari, Italy; 7 Department of Clinical, Special, and Dental Sciences, Marche Polytechnic University, 60126 Ancona, Italy; 8 Maria Cecilia Hospital, GVM Care & Research, 48033 Cotignola, Italy; 9 Department of Biomedical, Surgical and Dental Sciences, University of Milan, 20122 Milan, Italy

**Keywords:** atrial fibrillation, catheter ablation, mapping, intracardiac echocardiography, pulsed field ablation

## Abstract

**Background:** The VARIPULSE platform is an advanced Pulsed Field Ablation (PFA) system fully integrated with electro-anatomical mapping system, employing a variable loop circular catheter (VLCC) for atrial fibrillation (AF) ablation. The objective of the study is to assess for the first time the feasibility, safety, and procedural impact of AI (artificial intelligence)-assisted ICE (intracardiac echocardiography) mapping with the CARTOSOUND FAM Module compared with conventional electroanatomical mapping during PFA. **Methods:** In this retrospective, multicenter study, 157 consecutive patients undergoing PFA for paroxysmal or persistent AF were included. Patients were divided into two groups: ICE-guided cohort (n = 64) and non-ICE-guided cohort (n = 93). Propensity score matching (PSM) was used to adjust for baseline differences. **Results:** AI-assisted ICE mapping was feasible in all cases. Compared with conventional mapping, it significantly reduced LA (left atrium) mapping time (median 5 vs. 8 min; *p* < 0.001), LA dwell time (33.5 vs. 38.5 min; *p* = 0.001), and fluoroscopy time (7.5 vs. 14 min; *p* < 0.001). The total number of PFA applications was similar across groups (*p* = 0.136). No major adverse events occurred in either cohort during the procedure or within the first month of follow-up. **Conclusions:** AI-assisted ICE mapping using the CARTOSOUND FAM Module enables accurate anatomical reconstruction and significantly optimizes procedural efficiency in PFA. This approach supports further development toward radiation-sparing and potentially fluoroscopy-free PFA workflows. For the first time, it addresses a gap in the current evidence regarding the use of ICE in PFA, building on evidence already established for radiofrequency ablation procedures.

## 1. Introduction

Atrial fibrillation (AF) is the most common clinically significant cardiac rhythm disorder and remains a major cause of morbidity and health care burden worldwide. Over the past two decades, catheter ablation has progressively become the most effective rhythm-control strategy, with randomized trials consistently demonstrating superior outcomes compared with antiarrhythmic drug therapy in the treatment of AF [1,2].

In recent years, pulsed field ablation (PFA), based on irreversible electroporation, has emerged as a novel non-thermal energy source with unique myocardial selectivity. By applying high-voltage electrical fields to atrial myocytes, PFA induces membrane disruption and cell death while largely sparing adjacent non-cardiac structures [3]. Early clinical studies have demonstrated that PFA provides efficacy and overall safety profiles comparable to radiofrequency or cryothermal ablation, with a significantly lower risk of extracardiac injury [4,5,6].

The VARIPULSE™ platform is a novel PFA system, fully integrated with the CARTO electroanatomical mapping system, that utilizes short-duration, high-voltage bipolar biphasic pulses with a multi-electrode variable loop circular ablation catheter (VLCC). With the VARIPULSE platform, the standard procedural workflow includes 3D electroanatomical mapping of the left atrium (LA) with the VLCC prior to ablation [7].

More recently, a deep learning-based imaging algorithm integrated with intracardiac echocardiography (ICE) (CARTOSOUND FAM Module^TM^) has been developed and demonstrated to be both feasible and accurate in atrial fibrillation ablation using radiofrequency energy, enabling operator-independent LA reconstructions from ICE clips acquired in the right atrium and right ventricular outflow tract [8,9]. However, this algorithm has not yet been evaluated in the setting of PFA procedures.

The use of ICE as an adjunct tool in AF ablation procedures has consistently been shown in the literature to enhance procedural safety for both radiofrequency and PFA. Moreover, ICE contributes to improved workflow efficiency and supports the implementation of “zero-fluoroscopy” strategies [10,11]. In this study, we aimed to access the feasibility and procedural impact of this novel AI-assisted ICE mapping approach in patients undergoing PFA for AF with the VLCC.

## 2. Materials and Methods

### 2.1. Study Settings and Groups

We conducted a retrospective, observational, multicentre study to assess the feasibility and procedural impact of AI-assisted three-dimensional (3D) left atrial mapping using the CARTOSOUND™ FAM Module (Biosense Webster, Inc., Irvine, CA, USA, version 1) during PFA with the variable loop circular catheter (VLCC; Biosense Webster, Inc., Irvine, CA, USA). The study included the first 157 patients (61% paroxysmal, 39% persistent) who undergoing AF PFA using the VLCC across four Italian arrhythmology centres (University Hospital Ospedali Riuniti in Ancona, San Donato Hospital in Milan, Monzino Cardiological Center IRCCS in Milan and Miulli Hospital in Acquaviva delle Fonti).

Patients were divided into two groups. In the ICE-guided cohort (n = 64), 3D LA reconstruction was performed using an AI-assisted ICE mapping approach. In the non-ICE-guided cohort (n = 93), 3D LA reconstruction was conducted using the VARIPULSE catheter or high-density mapping catheters.

According to the European recommendations all individuals had undergone medical examination, standard 12-lead ECG, echocardiography and trans-oesophageal echocardiography when deemed appropriatev [12]. All clinical data were accurately collected for each patient, including cardiovascular risk factors, cardiac history, bio-impedance measurements, current therapy, and all procedural details. The study was performed respecting the institutional standards, national legal requirements, the Helsinki declaration for ethical standards, and the study protocol was approved by an institutional review board. Informed consent for participation was obtained from all subjects involved in the study.

### 2.2. The PFA VARIPULSE Platform

The VARIPULSE™ platform comprises the PFA VLCC and a proprietary multichannel PFA generator (Trupulse PFA Generator; Biosense Webster, Inc.; Irvine, CA, USA), both compatible with an electro-anatomical mapping system (CARTO3 System; Biosense Webster, Inc., Irvine, CA, USA). The Trupulse generator delivers short duration-high voltage bipolar biphasic pulses to the VLCC catheter. The VLCC transmits bipolar energy through multiple, brief pulses applied over a few seconds across the electrodes, without the need for an external patch. Each pulse consists of a proprietary waveform with an energy of 1800 V, featuring a predefined amplitude, pulse width, and duration measured in microseconds. The VLCC is an open-irrigated, 8.5F bidirectional catheter equipped with 10 electrode rings. It allows D-curve deflections of 180° to one direction and 90° to the other. The distal loop has an adjustable diameter ranging from 25 to 35 mm (at room temperature) ensuring optimal contact with a wide range of PV ostial and antral sizes. Each 3-mm electrode is individually irrigated via 10 irrigation holes using a constant flush of 4 mL/min during both mapping and ablation. The loop geometry is visualized within the CARTO3 system via three magnetic sensors embedded in the loop, allowing accurate spatial localization. As with other 3D magnetic sensor-enabled catheters, the VLCC can also generate an electro-anatomical 3D map to guide the handling of the catheter inside atrium chambers. For study procedures, the CARTO3 v8 system was used, incorporating the Tissue Proximity Indication (TPI) software module. TPI provides real-time feedback on catheter-tissue proximity based on impedance measurements from the VLCC catheter electrodes, indicating tissue contact on the electroanatomic map. When an electrode contacts tissue, the TPI algorithm detects it by identifying a rapid change from the baseline impedance (blood pool impedance) to the impedance associated with tissue contact.

PFA applications was delivered in a bipolar configuration between paired or adjacent electrodes to generate contiguous lesions beneath the catheter; any energy is delivered between proximal and distal electrode (electrode #1–electrode #10). By default, all 10 electrodes are active and ready to ablate once the generator is set up in ablation mode; however, the operator can manually deselect ablation mode in up to 4 electrodes. Once switched on the ablation, the generator will deliver a sequence of 3 applications (250 ms each) spaced out by a time interval of 10 s for a total PFA time of ~21 s; throughout the text, this sequence of three application is defined ablation [13].

### 2.3. Intracardiac Echocardiography and CARTOSOUNDFAM MAP Module

ICE was performed using an 8F SoundStar catheter (Biosense Webster, Inc., Diamond Bar, Inc., Irvine, CA, USA), introduced via percutaneous femoral venous access. The catheter is equipped with a linear-phased array multi-frequency transducer (5.5 to 10 MHz) and incorporates a 3D magnetic sensor compatible with the CARTO3 system. This configuration allows real-time visualization of the IE imaging plane synchronized with the mapping system to acquire intracardiac contours and generate a Fast-Anatomical Map. The AI-assisted ICE Module is a deep learning-based software integrated into the CARTO mapping system that automatically reconstructs detailed 3D left atrial anatomy from two-dimensional ICE clips acquired from the right atrium or right ventricular outflow tract, enabling operator-independent anatomical mapping prior to transseptal access. The mapping process using the AI-assisted ICE Module is detailed in the following section.

### 2.4. Ablation Procedure

Either general anesthesia or deep sedation were used according to the anesthesiologist and operator preference. Vascular access was obtained under ultrasound guidance to minimize peripheral vascular complications, and a diagnostic catheter was positioned within the coronary sinus. In ICE-guided cohort, an additional access was obtained to advance the SoundStar Catheter into the right atrium. In the non-ICE-guided cohort the use of ICE was determined at the operator’s discretion. Typically, ICE access was obtained via the left femoral vein to ensure the right leg remains available for the therapeutic catheter.

In the ICE-guided cohort, 3D LA anatomical reconstruction was performed using ICE imaging from the right atrium, and the mapping phase was completed prior to transseptal puncture to minimize sheath and catheter dwell time. Conversely, in the non-ICE-guided cohort, LA anatomical mapping was conducted using the VLCC or other high-density mapping catheters following transseptal access.

Transseptal puncture was performed under ICE and/or fluoroscopic guidance according to standard techniques [2,6]. LA access was achieved using a standard fixed sheath (SL0), which was subsequently exchanged over a guidewire for an 8.5F deflectable VIZIGO™ sheath (Biosense Webster, Inc., Irvine, CA, USA). The VIZIGO sheath is equipped with four distal electrodes that enable real-time visualization within the CARTO^®^ mapping system. Throughout the procedure, the sheath was continuously flushed with heparinized saline using a pressurized infusion system. Activated clotting time (ACT) was maintained at ≥350 s during LA dwell. To achieve this, an initial intravenous bolus of 8000–12,000 units of heparin was administered prior to transseptal puncture, followed by continuous infusion thereafter.

#### 2.4.1. Mapping Phase in ICE-Guided Cohort

In the ICE-guided cohort, prior to transseptal puncture, LA anatomy was reconstructed using the SoundStar™ catheter. Starting from the “home view” in the right atrium, the probe was gradually rotated clockwise toward the LA, automatically acquiring sagittal contours of the atrial anatomy. The procedure began with the probe positioned to visualize the left superior PV, where several image frames were acquired. The probe was then rotated clockwise to capture additional frames of the left inferior PV, followed by progressive rotation to acquire posterior wall contours until the right PVs were reached in the transverse plane. To obtain long-axis views of the right PVs, the probe tip was slightly deflected anteriorly or posteriorly as needed.

A total of approximately 30 contours were typically acquired: four for each PV, ten for the posterior wall, and four for the LA appendage. Special attention was paid to ensure that adjacent image slices were equitably spaced; if two consecutive slices appeared visually too distant, intermediate slices were obtained between them [14]. For enhanced visualization of the LA appendage and the ridge between the appendage and the left superior PV, the probe was deflected anteriorly from the home view, advanced into the right ventricular outflow tract, and then rotated clockwise to acquire the necessary views.

After completion of the contour acquisition phase, 3D reconstructions of the LA and adjacent anatomical structures—including the LA appendage, left and right superior pulmonary veins, and left and right inferior pulmonary veins—were generated using a deep learning-based algorithm integrated into the mapping system.

**Figure 1:**
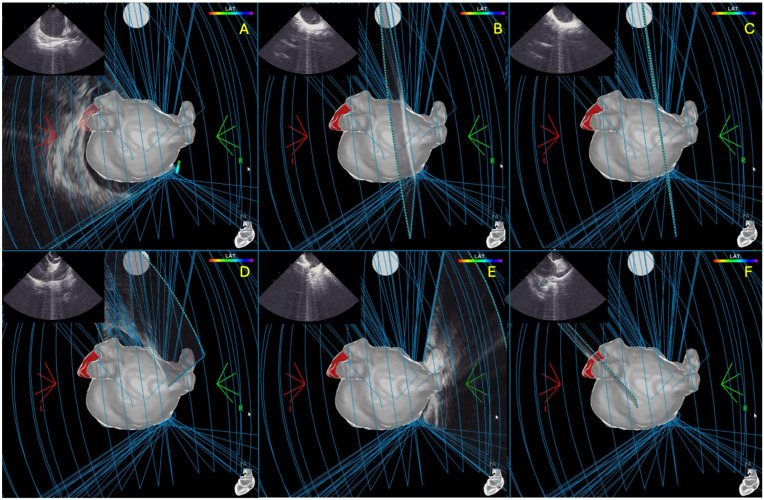
Progressive reconstruction of the left atrial anatomy using the CARTOSOUND FAM MAP Module, starting from the left superior pulmonary vein (**A**), moving through the left inferior pulmonary vein, posterior wall (**B**–**D**), right superior pulmonary vein, and right inferior pulmonary vein (**E**), until the reconstruction of the left atrial appendage (**F**).

Following the mapping phase, the VLCC was introduced through the sheath and advanced into the LA. Once positioned, the catheter loop was constricted to its minimum diameter (25 mm) and gently rotated within the chamber to ensure accurate calibration during TPI training. At this point, the system was fully configured and ready for pulsed field ablation (Figure 1).

#### 2.4.2. Mapping Phase in Non-ICE-Guided Cohort

Following the same precautions outlined above for the introduction and advancement of the VLCC, FAM-mapping was initiated after completing TPI training. To optimize catheter manoeuvrability and minimize unintended volume acquisition, the catheter loop was reduced to 25 mm before advancing it into each of the four pulmonary veins. The mapping of the LA body was subsequently completed to achieve a comprehensive virtual representation of the anatomy ready for the ablation phase.

#### 2.4.3. Ablation Phase

The ablation workflow was consistent across both study groups. For each PV, four ablations were performed—two with the catheter loop closed (25 mm) and two with the loop open (35 mm). PVI was initiated by positioning the VLCC with a closed loop at the PV ostium to deliver the first ablation. The second ablation was applied after slightly rotating the catheter to cover the gap between electrodes #1 and #10. The same approach was then repeated using the open loop configuration: the third ablation was delivered after repositioning the catheter more antrally, and the fourth ablation followed a slight rotation to close any remaining gap between the distal electrodes.

**Figure 2:**
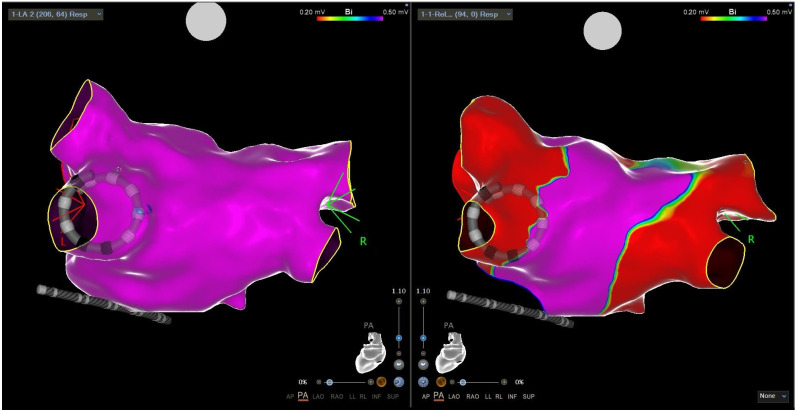
Three-dimensional reconstruction of the left atrium using the Varipulse catheter (**left**) and subsequent mapping after transcatheter ablation with PFA, showing evidence of pulmonary vein disconnection on voltage electroanatomical mapping (**right**).

For PVI-only procedures, a total of ≥16 ablations were recommended, corresponding to four complete applications per vein, creating two concentric wide-band lesions—one ostial and one antral. In patients with persistent atrial fibrillation, additional ablation sites were targeted at the operator’s discretion, which could include the posterior wall, superior vena cava, cavo-tricuspid isthmus, and anterior wall. On both the posterior and anterior atrial walls, contiguous ablation applications were delivered adjacently to ensure complete homogenization of the treated regions (Figure 2).

Following ablation, the elimination of pulmonary vein potentials was confirmed through mapping with the VLCC. Additional ablations were applied as necessary to address any residual conduction gaps. Isolation of the posterior wall and conduction block along additional ablation lines were verified using a rapid local activation time (LAT) map during coronary sinus pacing.

### 2.5. Study Endpoints

The primary endpoint of this study was to evaluate the feasibility of an AI-assisted, ICE-guided approach for reconstructing left atrial (LA) anatomy prior to ablation using the VARIPULSE™ system. This analysis aimed to determine whether ICE imaging could reliably generate anatomically accurate 3D maps suitable for guiding the ablation procedure.

The secondary endpoint was to assess the safety of LA mapping performed with ICE during the pre-ablation phase, verifying that the technique did not increase the risk of procedure-related complications.

Mapping time was calculated as follows: in the ICE-guided cohort, it was measured from the acquisition of the first ICE frame to the completion of the system-generated 3D reconstruction of the LA; in the non-ICE-guided cohort, it was measured from the introduction of the VARIPULSE catheter into the LA to the completion of the corresponding anatomical map.

Additional secondary endpoints included total procedure time, LA dwell time, and fluoroscopy time.

### 2.6. Statistical Analysis

Continuous variables were assessed for normality using the Shapiro-Wilk test. Data were expressed as mean ± standard deviation (SD) for normally distributed variables and as median with interquartile range (IQR: 25th–75th percentile) for non-normally distributed variables. Categorical variables were reported as counts and percentages. Group comparisons were conducted using the chi-squared test for categorical variables, the Student’s *t*-test for normally distributed continuous variables, and the Mann–Whitney U test for non-normally distributed continuous variables. A *p*-value of <0.05 was considered statistically significant.

To control for imbalance in baseline characteristics between study groups, propensity score matching (PSM) was performed (nearest neighbor method; caliper width: 0.25; no replacement) considering five variables (age, sex, paroxysmal AF, left atrial volume, extra-PVI lesion). The covariate balance after PSM was assessed by estimating standardized differences between groups, with a standardized difference >10% indicating significant imbalance between groups. To minimize potential confounding, age and sex were included to ensure comparability of baseline demographic features. Left atrial volume was selected due to significant baseline differences observed between groups. The type of atrial fibrillation (AF) was incorporated as it demonstrated a near-significant trend and constitutes a key determinant in both the pathophysiological profile and interventional strategy for AF ablation. Lastly, ablation of extra-pulmonary foci was considered, as this variable also differed significantly at baseline.

Statistical analyses were performed using the Statistical Package for the Social Sciences (SPSS^®^, IBM Corp., Armonk, NY, USA) and R statistical software (R Foundation for Statistical Computing, Vienna, Austria); *p* values < 0.05 were considered statistically significant.

## 3. Results

### 3.1. Patient Population

Clinical and echocardiographic characteristics, as well as pre-procedural AADs, are summarized in Table 1.

To homogenize the two cohorts, PSM was performed based on six variables: age, sex, type of AF, LA volume, and presence of extra-PVI lesions. PSM yielded a total of 100 matched patients (50 per cohort). Patients were also matched with respect to extra-PVI ablations, as these procedures were performed across different centers according to the local standard of care and the decision to ablate the anterior wall or other structures was made at the discretion of each center.

**Figure 3:**
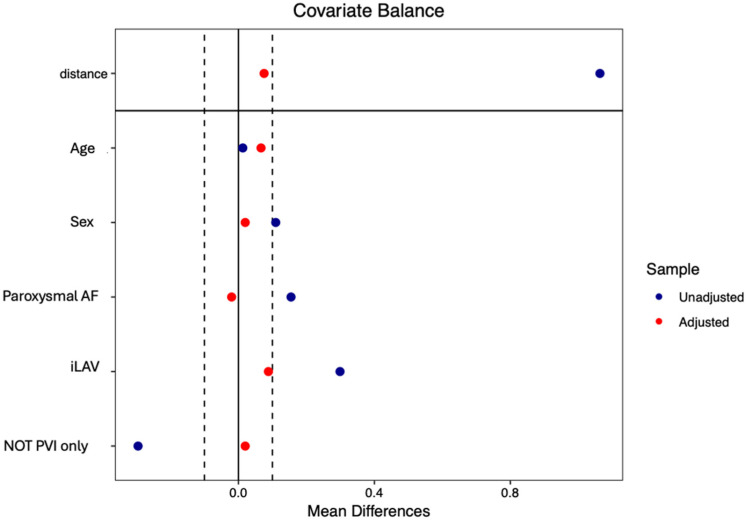
Propensity score matching of patients from the two cohorts: ICE-guided cohort and non-ICE-guided cohort, matched for age, sex, indexed left atrial volume (iLAV), type of atrial fibrillation (AF), and ablations beyond the pulmonary veins (NOT PVI only).

Baseline clinical and echocardiographic characteristics were comparable between the two cohorts, except for paroxysmal AF, which tended to be more frequent in ICE-guided cohort, and LA volume, which was significantly larger in ICE-guided cohort. After PSM, both the prevalence of paroxysmal AF (n = 38 (76%) for ICE-guided vs. n = 39 (78%) for non-ICE-guided cohort; *p* = 0.812) and LA volume (36 (11.6) mL/m^2^ vs. 34.8 (9.6) mL/m^2^; *p* = 0.212) were balanced between the two cohorts [Figure 3].

### 3.2. Feasibility and Safety

In ICE-guided cohort, LA 3D reconstructions using the ICE-based workflow and the AI-assisted ICE Module was feasible in each patient. Only 4 out of 64 (6%) patients required, after reconstructing the map with the AI-assisted ICE Module, the acquisition of new images using ICE to complete the map, as certain portions were missing (in 2 cases, the right inferior pulmonary vein; in 1 case, the left superior pulmonary vein; and in 1 cases, the right superior pulmonary vein).

No major adverse events occurred during the procedure and until the end of the first month of follow-up, irrespective of the study groups (*p* = 1; 95% CI, −5.66–3.97% for non-matched cohorts; *p* = 1; 95% CI, −7.13–7.13% for matched cohorts). There were no cases of cardiac tamponade, atrio-esophageal fistula, pulmonary vein stenosis, or pericardial effusion. Regarding minor complications, a single vascular complication (arteriovenous fistula) occurred, which was managed conservatively with compression (*p* = 0.742; 95% IC −7.74–7.23% for non-matched cohorts; *p* = 1; 95% IC −10.15–10.15% for matched cohorts).

### 3.3. Procedural Data

The procedural data from both the overall and the propensity-matched cohorts are summarized in Table 2.

Statistically significant differences observed in the overall cohort remained consistent in the matched cohort, except for AW ablation, whose baseline difference was eliminated through PSM (*p* = 0.081).

Although the skin-to-skin procedural times were comparable between the two groups (ICE-guided cohort 65 [60–77] min; non-ICE-guided cohort 60 [60–60] min; *p* = 0.424), patients in the ICE-guided cohort showed significantly shorter LA mapping times (5 [4–5] vs. 7 [6–7] min; *p* < 0.001), LA dwell times (30.5 [25.5–35] vs. 35 [28–35] min; *p* = 0.035), and fluoroscopy times (7 [6–9] vs. 14 [13–14] min; *p* < 0.001) compared to those undergoing VARIPULSE electro-anatomic mapping, both in the overall study population and in PSM patients (Table 2).

As expected, the points collected with the VARIPULSE/mapping catheter before the first ablation are significantly higher in non-ICE-guided cohort (401 [190–725] vs. 1970 [1110–1901]; *p* < 0.001). In the ICE-guided cohort, a statistically significant higher presence of common trunks was also identified (25 (39%) vs. 7 (8%); *p* < 0.001).

Finally, in the propensity-matched cohort, a statistically significant difference was observed in the total number of applications (64.5 [57–87.7] in ICE-guided vs. 54 [51–54] in non-ICE-guided cohort; *p* = 0.004).

## 4. Discussion

### 4.1. Main Findings

This study represents the first multicenter experience evaluating AI-assisted LA mapping using ICE technology in the context of PFA. By directly comparing this novel workflow with conventional electroanatomical mapping using the VARIPULSE catheter, our findings provide early real-world evidence of its feasibility, safety, and potential procedural advantages. The main findings of our study can be summarized as follows:

- The 3D reconstruction of the LA, performed using both the new AI-assisted ICE Module and electroanatomic mapping with high-density mapping catheters, was found to be feasible. Both techniques enable a clear anatomical reconstruction of the LA with short mapping times.
- Although both LA reconstruction techniques demonstrated short LA dwell time, these times were significantly shorter in ICE-guided cohort. Additionally, ICE-guided cohort was associated with approximately half the radiation exposure compared to non-ICE-guided cohort. These advantages were confirmed even after propensity matching, employed to account for potential heterogeneity in baseline clinical characteristics of the patients.
- In a large real-world cohort, despite these being the initial cases performed with this new technology, both techniques exhibited a high safety profile, with a major complication rate of 0% and a minor complication rate of less than 1% (single non-surgical vascular complication).

### 4.2. Procedure Efficiency and AI-Assisted ICE Module

Although the patients analyzed in this study were among the first to undergo ablation with the novel PFA technology, the procedural metrics, including fluoroscopy time, mapping time, and LA dwell time, were notably low. These results were comparable to, and in some cases lower than, those previously reported in the literature for similar PFA procedures [13,14].

When compared with studies utilizing alternative energy sources such as radiofrequency or cryothermal ablation, the procedural durations observed in this cohort were similar or shorter. Likewise, when contrasted with other widely used PFA technologies, such as those employing a five-spline catheter, procedural times remained comparable. Importantly, the VARIPULSE™ platform offers the potential to further reduce fluoroscopy exposure, owing to its seamless integration with the electroanatomical mapping system, although we did not have the follow-up data available to directly compare these techniques [15,16].

Further subgroup analysis based on the LA mapping method demonstrated that the use of the AI-assisted ICE module was associated with additional reductions in fluoroscopy time, LA mapping time, and a marked decrease in LA dwell time. The combined use of electroanatomical mapping and ICE-derived anatomical reconstruction contributed significantly to minimizing fluoroscopy exposure. Notably, fluoroless ablation protocols using this technique have also been described in the literature [17].

AI-assisted ICE mapping enabled accurate LA anatomical reconstruction, resulting in shorter LA dwell times and a substantial reduction in fluoroscopy exposure. These procedural benefits were achieved despite implementation during the initial learning phase, suggesting that further optimization is likely with increasing operator experience. The reduction in LA dwell time remained statistically significant between the two study cohorts after patient matching; however, the magnitude of this reduction was modest (*p* = 0.035), as LA mapping represents only a small fraction of total LA time, which is largely determined by ablation duration.

The integration of electroanatomical mapping with AI-guided ICE imaging also simplifies AF ablation by avoiding redundant or overlapping energy applications, identifying untreated areas, and assessing catheter–tissue contact through the TPI module. Moreover, intracardiac echocardiography provides a major advantage in evaluating contact quality, ensuring circumferential energy delivery around each PV without residual gaps. It also facilitates precise positioning and delivery of contiguous lesions at the level of extrapulmonary triggers and allows for real-time verification of lesion continuity [7].

**Figure 4:**
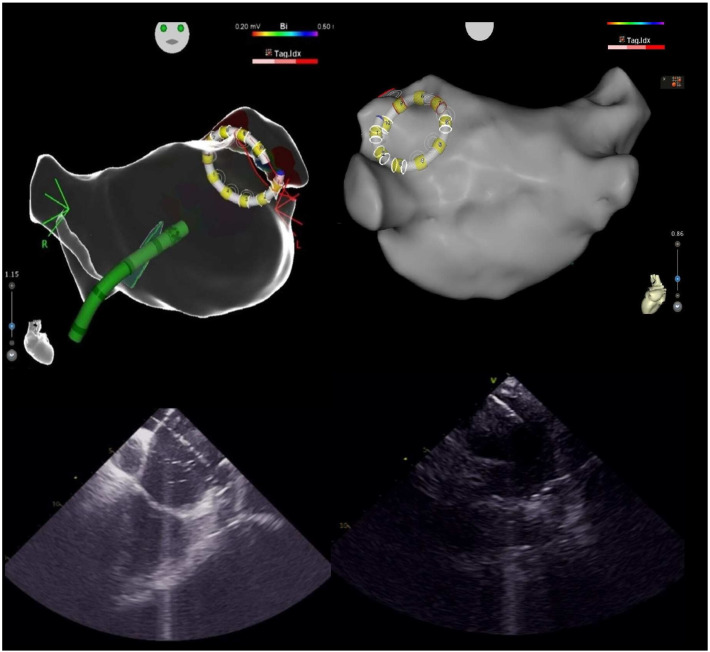
The top section shows map views reconstructed using the CARTOSOUNDFAM MAP Module with the VARIPULSE catheter introduced at the level of the left upper pulmonary vein, while the bottom section presents the ICE visualization of the same moment. The image on the right highlights the presence of a left common trunk with VARIPULSE catheter in ostial position, clearly visible also in the echocardiographic images.

Previous studies have shown that even minor gaps between the catheter and the atrial wall can lead to significant attenuation of electrograms while still permitting tissue recovery. Therefore, achieving optimal electrode–tissue contact prior to energy delivery is essential. Traditional analyses of electrogram parameters, including amplitude and frequency, have demonstrated correlations with contact quality [18,19,20]. In addition, advanced mapping system algorithms that assess tissue geometry and proximity further enhance the precision of ablation when using the VLCC catheter [20,21] (Figure 4).

However, no significant reduction in total procedural time was observed. Despite the significant decrease in left atrial dwell time, this finding may be explained by the fact that total procedural time includes various technical steps and procedural phases -such as vascular access, catheter positioning, transseptal puncture, and remapping- that similarly affect both groups, thereby resulting in no significant difference in overall procedural duration.

### 4.3. Safety

The findings of the present study are consistent with prior research on this novel VLCC, which have demonstrated that the PFA system produces transmural lesions without causing collateral damage to adjacent structures. Previous studies have shown that the new VARIPULSE platform and VLCC are cardio-selective, avoiding major adverse events such as atrio-esophageal fistula, pulmonary vein stenosis, or phrenic nerve paralysis [13,14]. These outcomes were further validated in a porcine model, in which lesions were both effective and durable, achieving substantial depth without injuring phrenic nerve, esophagus, or pulmonary veins [16,17,18,19,20].

The favorable safety profile extends, within the limitations of the study’s sample size and the short 1-month follow-up, to extrapulmonary ablation sites including the posterior wall, roof, anterior wall of the LA, mitral and cavotricuspid isthmus, and superior vena cava. The study demonstrates that energy delivery to these targets is feasible and safe, without evidence of collateral damage or procedure-related one-month adverse events. Notably, no cases of perforation, tamponade, pericardial effusion, post-ablation pericarditis, or injury to surrounding structures were observed. Additionally, no TIAs or strokes occurred, including during extra-PV ablations. During ablation at the mitral and cavotricuspid isthmus, prophylactic intravenous nitrates were not administered, and no ECG abnormalities indicative of coronary spasm were detected.

The use of intracardiac echocardiography (ICE) enabled the statistically significant identification of common venous ostia (25/64 [39%] vs. 7/93 [8%]; *p* < 0.001), consistent with previous reports [22,23]. ICE provides enhanced visualization and a detailed assessment of cardiac anatomy, including the pulmonary veins, thereby facilitating more targeted lesion formation. It allows for a comprehensive evaluation of common ostia and precise visualization of catheter position within the ostium, helping to prevent lesions that are either excessively ostial or insufficiently antral. This capability contributes to an improved safety profile and may enhance the efficacy of lesion formation.

Despite the high median number of energy applications in the LA (median 66 [IQR 56–96] in the ICE-guided cohort; median 60 [IQR 54–87] in the non–ICE-guided cohort), no cases of acute renal failure requiring intervention were reported. The inspIRE study demonstrated that performing fewer than 16 ablations (48 applications) nearly doubled recurrence rates [21]. Conversely, excessively high ablation doses may induce peripheral tissue stunning; preclinical dose–response studies indicate that high-dose applications can result in temporary regression of lesion volume, as assessed histopathologically over 30 days, suggesting transient effects at lesion peripheries [20].

Although these safety outcomes are encouraging, this study represents one of the first clinical investigations of this PFA system. Consequently, it remains essential to maintain procedural vigilance and adhere to optimized workflows to minimize the risk of complications. The ability to generate operator-independent 3D maps provides distinct procedural advantages, including optimized catheter navigation, reduced intra-atrial manipulations, and the potential to streamline training for less experienced centers. Moreover, minimizing radiation exposure aligns with contemporary trends toward “zero-fluoroscopy” strategies, which is particularly relevant for younger patients and high-volume operators.

### 4.4. Study Limitation

The main limitations of the study are (1) the small retrospective sample size included in the study, (2) the limitation of sample selection to highly experienced arrhythmia centers with a high volume of cases and the performance of extra-PV lesions was left to the operator’s discretion, and (3) the lack of post-ablation follow-up preventing us to describe post-ablation outcomes of this novel technique and that restrict the clinical applicability of the conclusion.

The small sample size, retrospective design, and the inclusion of patients exclusively from high-volume, experienced centers may introduce selection bias, limit the generalizability of the findings to broader populations and lower-volume centers, and restrict reproducibility on a larger scale. Additionally, the absence of medium- to long-term follow-up precludes assessment of procedural efficacy over time, particularly regarding whether the observed improvements in procedural metrics translate into sustained clinical benefit.

## 5. Conclusions

This study demonstrates that the AI-assisted ICE-guided reconstruction of the left atrial anatomy using the AI-assisted ICE Module is both feasible and safe, enabling reliable anatomical mapping to guide pulsed field ablation without increasing procedural risk. Compared with conventional electroanatomical mapping, this approach achieved meaningful reductions in left atrial dwell time and fluoroscopy exposure, thus highlighting the potential of AI-assisted ICE mapping to streamline PFA workflows and support the development of fully fluoroscopy-free ablation strategies.

## Figures and Tables

**Table 1:** Clinical, echocardiographic characteristics and pre-ablation AAD therapy. Abbreviations: AAD, antiarrhythmic drug; AF, atrial fibrillation; BMI, body mass index; CHA2DSVA, Congestive heart failure, Hypertension, Age 75 years or older, Diabetes mellitus, previous Stroke or transient ischaemic attack, Vascular disease, Age 65–74 years old; COPD, chronic obstructive pulmonary disease; EGFR: estimated glomerular filtration rate; ICE, intracardiac echocardiography; ILAV, indexed left atrial volume; IM, mitral insufficiency; IQR, interquartile range; LVEF, left ventricular ejection fraction; N, number; NOAC, non-vitamin K antagonist oral anticoagulants; TIA, transient ischemic attack; TAPSE, tricuspid annular plane systolic excursion.

	ICE-Guided Cohort (N = 64)	Non-ICE-Guided Cohort (N = 93)	*p* Value	Matched ICE-Guided Cohort (N = 50)	Matched Non-ICE-Guided Cohort (N = 50)	*p* Value
Age—Median [IQR]	62 [59.5–69]	62 [58–67]	*p* = 0.453	62 [58–69]	61.5 [55.3–61]	*p* = 0.347
Male Sex—N (%)	49 (77)	61 (66)	*p* = 0.194	35 (70)	34 (68)	*p* = 0.829
Paroxysmal AF—N (%)	45 (70)	51 (55)	*p* = 0.074	38 (76)	39 (78)	*p* = 0.812
First therapy AF ablation—N (%)	17 (27)	14 (15)	*p* = 0.115	14 (28)	8 (16)	*p* = 0.148
Months AAD Therapy—Median [IQR]	12 [6.5–15]	13 [6–36]	*p* = 0.082	12 [6–36]	16.5 [7–16]	*p* = 0.276
Previous Ablation—N (%)	9 (14)	25 (27)	*p* = 0.085	8 (16)	16 (32)	*p* = 0.061
CHA2DSVA—Median [IQR]	1 [1–2.25]	1 [0–2]	*p* = 0.459	1 [0–2]	1 [0–1]	*p* = 0.711
Hypertension—N (%)	36 (56)	56 (60)	*p* = 0.740	26 (52)	30 (60)	*p* = 0.420
Dyslipidaemia—N (%)	36 (65)	41 (44)	*p* = 0.182	28 (56)	21 (42)	*p* = 0.194
Diabetes Mellitus—N (%)	8 (13)	6 (7)	*p* = 0.307	6 (12)	3 (6)	*p* = 0.480
EGFR < 45 mL/min/m^2^—N (%)	1 (2)	5 (5)	*p* = 0.423	1 (2)	3 (6)	*p* = 0.208
Smoke—N (%)	7 (11)	18 (19)	*p* = 0.232	7 (14)	11 (22)	*p* = 0.299
COPD—N (%)	2 (3)	6 (7)	*p* = 0.574	2 (4)	2 (4)	*p* = 0.400
Vascular Disease/CAD—N (%)	8 (13)	9 (10)	*p* = 0.766	7 (14)	7 (14)	*p* = 1
Osas—N (%)	11 (17)	14 (15)	*p* = 0.890	8 (16)	6 (12)	*p* = 0.564
Stroke/TIA—N (%)	1 (2)	3 (3)	*p* = 0.892	1 (2)	2 (4)	*p* = 0.558
Tachycardiomyiopathy—N (%)	4 (6)	1 (1)	*p* = 0.176	4 (8)	1 (2)	*p* = 0.169
BMI—Median [iqr]	26.6 [25.4–28.4]	27.9 [24.6–31]	*p* = 0.197	26 [25–28]	25.5 [23.3–25]	*p* = 0.728
LVEF %—Median [IQR]	60 [55.7–61]	60 [56–63]	*p* = 0.202	60 [55–60]	60 [56–60]	*p* = 0.390
Severe IM—N (%)	0 (0)	0 (0)	*p* = 1	0 (0)	0 (0)	*p* = 1
ILAV (ml/m2)—Mean (st)	38.4 (12.7)	34.6 (9.3)	*p* = 0.009	36 (11.6)	34.8 (9.6)	*p* = 0.212
TAPSE (mm)—Median [iqr]	22 [20–24]	22 [20–24]	*p* = 0.689	22 [20–24]	22 [20–22]	*p* = 0.617
Pre-Ablation AAD therapy - Beta-Blocker—N (%) - Flacainide/Propafenone—N (%) - Amiodarone—N (%) - NOAC—N (%)	39 (61) 23 (36) 9 (14) 55 (86)	42 (45) 37 (40) 23 (25) 80 (86)	*p* = 0.075 *p* = 0.748 *p* = 0.153 *p* = 0.826	27 (54) 21 (42) 5 (10) 41 (82)	21 (42) 19 (38) 12 (24) 38 (76)	*p* = 0.230 *p* = 0.683 *p* = 0.064 *p* = 0.461

**Table 2:** Procedural data. Abbreviations: ABL., ablation; AF, atrial fibrillation; AW, anterior wall; CTI, cavo-tricuspid isthmus; ICE, intracardiac echocardiography; IQR, interquartile range; LA, left atrium; MI, mitral isthmus; N, number; PV, pulmonary vein; PVI, pulmonary vein isolation; PW, posterior wall; SVC, superior vena cava; 3D, 3-dimensional.

	ICE-Guided Cohort (N = 64)	Non-ICE-Guided Cohort (N = 93)	*p* Value	Matched ICE-Guided Cohort (N = 50)	Matched Non ICE-Guided Cohort (N = 50)	*p* Value
ICE—N (%)	64 (100)	12 (13)	*p* < 0.001	50 (100)	11 (22)	*p* < 0.001
3D Anatomical Mapping - Varipulse—N (%) - Pentaray—N (%) - Octaray—N (%) - ICE Soundfam—N (%)	0 (0) 0 (0) 0 (0) 64 (100)	93 (100) 0 (0) 0 (0) 0 (0)	*p* < 0.001 *p* = 1 *p* = 1 *p* < 0.001	0 (0) 0 (0) 0 (0) 50 (100)	50 (100) 0 (0) 0 (0) 0 (0)	*p* < 0.001 *p* = 1 *p* = 1 *p* < 0.001
Pre-ABL. Point—Median [IQR]	401 [171–725]	2100 [1221–2834]	*p* < 0.001	401 [190–725]	1970 [1110–1901]	*p* < 0.001
- Left Common PV—N (%) - Right Common PV—N (%)	25 (39) 0 (0)	7 (8) 2 (2)	*p* < 0.001 *p* = 0.147	21 (42) 0 (0)	4 (8) 1 (2)	*p* < 0.001 *p* = 0.315
PW Ablation—N (%)	22 (34)	39 (42)	*p* = 0.430	17 (33)	10 (20)	*p* = 0.115
Extra PV/PW Ablation - MI—N (%) - CTI—N (%) - AW—N (%) - SVC—N (%)	3 (5) 5 (8) 3 (5) 0 (0)	2 (2) 3 (3) 16 (17) 2 (2)	*p* = 0.374 *p* = 0.360 *p* = 0.018 *p* = 0.147	2 (4) 6 (12) 2 (4) 0 (0)	2 (2) 2 (4) 7 (14) 2 (4)	*p* = 1 *p* = 0.140 *p* = 0.081 *p* = 0.153
N° TOT Application—Median [IQR]	66 [56–96]	60 [54–87]	*p* = 0.136	64.5 [57–87.7]	54 [51–54]	*p* = 0.004
Complete PVI During Remap—N (%)	62 (97)	93 (100)	*p* = 0.165	52 (100)	52 (100)	*p* = 1
Mapping Time (Min)—Median [IQR]	5 [4–5]	8 [6–11]	*p* < 0.001	5 [4–5]	7 [6–7]	*p* < 0.001
Time in LA (Min)—Median [IQR]	33.5 [26–35]	38.5 [30–46]	*p* = 0.001 [171–725	30.5 [25.5–35]	35 [28–35]	*p* = 0.035
PROC. TIME (Min)—Median [IQR]	65 [60–75]	60 [60–75]	*p* = 0.535	65 [60–77]	60 [60–60]	*p* = 0.424
Fluoro Time (Min)—Median [IQR]	7.5 [6–9]	14 [9.2–18.3]	*p* < 0.001	7 [6–9]	14 [13–14]	*p* < 0.001
AF Recurrence During Hospitalizations—N (%)	4 (6)	4 (4)	*p* = 0.860	4 (8)	2 (4)	*p* = 0.400
Minor Complication—N (%)	1 (2)	2 (2)	*p* = 0.742	1 (2)	1 (2)	*p* = 1
Major Complication—N (%)	0 (0)	0 (0)	*p* = 1	0 (0)	0 (0)	*p* = 1

## Data Availability

The data presented in this study are available on request from the corresponding author due to privacy and legal reason.
